# Why This World Is like a Hospital

**Published:** 1913-12-27

**Authors:** Bernard Vaughan


### WHY THIS WORLD IS LIKE A HOSPITAL.

### By the Rev. Father Bernard Vaughan, S.J.

**Being his Address to the League of Mercy at St. James's Palace.**

Your Royal Highness, Your Serene Highness, My Lords, Ladies and Gentlemen,—This suffering world in which we live has been compared to a stage, to a school, to a drill-ground, to a battle-field, and to many other institutions. To-day I will compare it, with your kind indulgence, to a hospital. I say this world is like unto a hospital. No one enters it but you can say to that one, if you live on this planet you will become acquainted with suffering and with sorrow, and finally you will meet death. I may say, then, that this world is not unlike a hospital. Indeed, I would call it to-day—not usually, but we are met here to-day rather in the minor key, looking to the sorrows of our poor suffering brothers and sisters—I will say it is like a hospital of incurables. Anyhow, I have lived many years on this planet, and I have never seen anyone go out of it alive. (Laughter.) I am told, in your very instructive literature, that one in every four Londoners benefits by the hospitals of this country. The hospitals are our pride and our glory, because they are the voluntary contributions of our hearts. (Applause.) Ladies and gentlemen, I am proud of our hospitals, and I think there are more than a hundred of them in London dealing, from cancer down to measles, with every infirmity poor flesh is heir to. These hospitals are your special care; they are under your patronage and under your love, your guidance and care. You go forth to draw into the net, which you are going to let down deeper from all accounts, contributions for this splendid work, of which every Englishman throughout the Empire is so proud, under the voluntary system of our hospitals. I assure you that from all sorts and conditions of men and women from different parts of the world I hear expressions of admiration and expressions of delight at our method in regard to hospitals. They tell me it is the last note, these most up-to-date institutions, our hospitals, that it is a bright feature in our country, that the staffs of nurses are pre-eminently efficient, and that all cases are handled in a masterly way by our surgeons and doctors. How proud I am then, Sir, to stand upon this platform and to advocate the cause which her Royal Highness and you, Sir, have so deeply at heart.

### Animal Dances.

I know that in a day when we are so much given to the culture of animal pleasures and animal dances, and to the worship of the flesh, it is very unpopular to speak about this world as a hospital for cripples and for incurables. But I think that we, with our virile sense of duty to our country, should take matters of fact as they are, and not as we would like them to be. (Hear, hear.) And I think that we have not, after all, anything so very much to fall down and worship in the poor human body, which is subject to so many ills, and which, unless taken the most precious and constant care of, is a horror to ourselves and to those who are nearest to us. It is much better to look at these things in the face when we are dealing with ourselves. I say, then, that the great note of distinction between this land and that land beyond the stars to which you and I are going is just this: that we are told here—very different from there—when we cross the threshold yonder that death and sorrow shall be no more, the former things shall have passed away. And then we have the promise—then, but not till then, not till we have crossed the border-line—then, gentlemen and ladies, not till then, the Great Physician of Souls will wipe every tear from our eyes. So let us look at this world not as a terminal arrival but as an outward voyage; not as a play but as a rehearsal, or, if you like, a prologue. Here we have to learn, here we have to train, here we have to be of use to one another, to be always looking to the interests of each other, always to be alert, alive, active, live wires. That is a sign that we have to be energetic and at work.

But when we have crossed the border, when we have gone to the land beyond, ladies and gentlemen, you and I, when we ring the bell of that great House, we shall be alone. You will not find a servant's bell and a tradesman's bell; you will find when you arrive there that all visitors come to stay. You may, then, when you cross that threshold, say, I am on strike for all eternity, for I have done all my work below.

### The Great Example.

We have, I think, on an occasion like this, after the official work which has been so satisfactorily and efficiently done, need to take away with us some thought which we may interpret into our life, and may weave into the very fibre of our being. And therefore I would like to say to you, with your kind indulgence, that we may learn from Him who is the greatest pattern of all virtue, and as the great historian reminds us, is the greatest incentive to its practice, we may learn from Him Who walked our earth how to look at poor, suffering humanity. We are told He saw the multitude and had compassion on them, and He broke bread and gave it to them. He opened His eyes to see, He opened His heart to feel, and He opened His hand to give. And I think you and I can learn no better lesson this afternoon than this: to look at life and to look more especially at our suffering brothers and sisters, particularly those lower down the social ladder, and to see their lives. You cannot get to understand the poor or the sick by reading the most eloquent books. You must go, as they say in America, "right in"; you must get "right in"; you must see their lives. I remember the last time I was with Pope Pius X.; as I was leaving him I said, "Send the message to my poor brothers in Commercial Road, London." He said, "Look at the charity and patience of the poor; it astonishes me daily more and more." (Hear, hear.) Ladies and gentlemen, you know sometimes we think poverty is such a terrible thing. And it is, when you want more; and as a rule nobody has got enough while somebody else has got more. But it is not so among my toiling brothers. They have got a very bright life of their own if they have got some little to go on with. When they are in health they are no slaves to tradition; they are not bound up by the conventionalities of life; they are not driven here and then driven there, and then driven back again, like some who move in sets where they are told almost what to do.

### The Poverty of London.

But the poor man is always hungry. the poor man can always sleep; he has an appetite for anything that he can get hold of, and if he has got a few pence he can make a very savoury meal and relish it thoroughly. And he can get out into the world and see all that other folk are doing. The other day I was giving a lecture in a theatre, and three or four poor girls came to me after. And they wanted me to sign my name on their bill. I said to one girl, "What are you doing?" She said she was tacking and seaming and doing a lot of things to mantles. "What do you get a week?" "Four and threepence, from 8 in the morning to 6.30 at night." Another girl got 6s. 3d., and the one between 5s. These girls were as happy and bright as could be. I hung down my head to think. "You don't get a penny an hour for your work in a country where we are saying 'Britons never shall be slaves!'" We are nothing else. (Hear, hear.) They were so happy; they did not complain, and I did not like to strike a note against the employers, though I think the very first draw upon any industry should be to pay those who produce it. But many people treat those they employ like they treat a hen; they get the egg but forget the hen. Ladies and gentlemen, what a fine little example was this! And I can go among the poor people, our suffering brothers and sisters, and find them always bright and cheery and hopeful as long as they are healthy. Of course, they cannot expect to be always supremely happy. I remember a Nonconformist minister, a friend of mine, was trying to impress on a meeting one day that we are not sent here for happiness, we are sent here to do our duty; and he asked if they had known anyone supremely happy, or read of anyone who was supremely happy. There was no response, and at last he ventured the silly remark, "Did you ever hear of anyone supremely happy?" Then it was that a lady rose from her seat and explained, "From all accounts my husband's first wife was supremely happy." (Laughter.) But we cannot all be husbands' first wives. And so I say, ladies and gentlemen, we must go and open our eyes and see. And it is so splendid to know what is actually going on and how people live; how they bear the burden of life, and how they come forth from it. Then, if we can open our eyes to see, we shall soon open our hearts to heal, and especially those of us who think that we have some great trial. I think there is nobody has a trial, but feels it is perhaps the supremely greatest in the world, and that trial is almost hugged. The Cross is taken up, and not set against the Master's on Calvary, but taken round the parish to show what we have. How does it come to me? Take your cross into some fever ward, take it into some hospital, or infirmary, or home, and set it beside the bed of the sufferer. You will come away with some sense of proportion; you will feel "Though mine is great, her's is greater." We can grow fine character under it, and that is what we want—character building out of these raw materials which lie around us. Ladies and gentlemen, to see the suffering, of the poor especially, because the poor are so incapable of looking after a sick child or a sick man or woman, is a discipline.

### A Grand Work.

I have been into sick rooms and taken off linseed poultices which were stiff and cold as a corpse, having been on twenty-four hours. That cannot help anybody. I know another instance, of a woman in Lancashire whose husband was ill. And the doctor called next day, and said, "Mrs. Walmsley, and how is our patient?" "I don't know how it is going to end, which road it's going to be." "Have you followed out my prescriptions?" "Well, doctor, I think we have, in the main, but it was not likely I was going to call you in and then find it was all wasted. We had no thermometer, doctor, so we borrowed Mrs. Jason's barometer." "What did you do?" "He had it on his stomach, but he complained of the weight, and then I sat on it. After a quarter of an hour I looked at it, and it pointed to 'Very dry,' so I gave him a drink." (Laughter.) There you see how life goes along. They are not capable of looking after the sick, not even their little children, and that is the reason we have such a mortality among the little ones. We come as the saviours of our broken brethren, especially of those who are in the arena, and I think it is a grand work; it is a grand work for ourselves, and it is a grand work done for others. It is also a splendid example to give to our people.

Lastly, we open our hearts, and then the hand opens. Why is it we have not fifty thousand pounds a year, instead of less than twenty thousand, in this League of Mercy—this League of Mercy which is twice blessed, for it blesseth him that gives and him that takes? Because I do not think the eyes have been opened sufficiently; the heart has had no opportunity. If it had, the hands would give out what is needed by the Treasurer. Ladies and gentlemen, measure thy life by loss, and not by gain; not by the wine drunk, but by the wine poured forth; for love serveth constantly in love's sacrifice, and whoso loves the most has most to give, and whoso suffers most has most to love.

### An Object Lesson.

Before resuming my chair, I must take this opportunity of saying something I feel so much. You know I am immensely pleased to be on this platform, because where there are religious principles at stake we have to run the walls up into the blue vault, and hold our own, even if we are to be crushed. But where principle is not at stake we should keep those walls so low as to be able, with love and gladness, to shake hands with the man on the other side. To-day you have let me come forth, not merely as a minister of religion, but to come forth as a subject of my King and Queen, as an Englishman. And I never allow anyone to stand between me and my King and Queen or my country. (Applause.) And I wish to say to-day how grateful I am to the Committee for allowing me to unburden my heart, which is so often pent up from want of opportunity of speaking to those who are not of my communion. Ladies and gentlemen, do you want an object-lesson to-day as an example of what I say, with eyes open to see, and a heart open to feel, and a hand open to give? I see beautiful examples of these virtues in our King and Queen—King George and Queen Mary. (Applause.) If you want an object-lesson, you have it at hand; you can see it every day; you can read it in the story of their lives. Their eyes are open to see their countrymen and countrywomen. The King is an eye to the blind, a foot to the lame, a hand to his brother, his people. You will find them in the hospitals, you will find them in the cottages, you will find them among the people. They are going to get first-rate knowledge, and their hearts are accordingly attuned to meet and sympathise with the suffering and the sorrowful. They have gone to the furthest, and come back to the nearest, and everywhere they have their eyes open to see, their hearts open to feel, and their hands, with the poor little gift offered them by the people, dole out until there is hardly anything left. I wish they had hundreds of thousands a year more to dispose of. But, in the meantime, we may look at their example, and thank God for their beautiful social and domestic lives, and so our prayer for our King may be—

"Happy and glorious,
Long to reign over us,
God save the King!"

(Applause.)

